# Supplementary material for: Prognostic analysis of very early onset pancreatic cancer: a population-based analysis
Source: PeerJ. 2020 Feb 10;8:e8412. doi: 10.7717/peerj.8412 (PMC7017800; doi:10.7717/peerj.8412)
Supplement: Table S1 [file peerj-08-8412-s001.docx]

**Supplemental Table 1.** **The demographic and treatment data of the whole cohort**

| Characteristics | No. of patients | % |
| --- | --- | --- |
| **Age (Years)** |  |  |
| <45 | 1386 | 2.5% |
| 45-59 | 13200 | 23.9% |
| 60-69 | 16919 | 30.6% |
| 70-79 | 15175 | 27.4% |
| >79-- | 8638 | 15.6% |
| **Sex** |  |  |
| Male | 28133 | 50.9% |
| Female | 27185 | 49.1% |
| **Race** |  |  |
| Caucasian | 44115 | 79.7% |
| African American | 6729 | 12.2% |
| American Indian/Alaska Native | 300 | 0.5% |
| Asian or Pacific Islander | 4174 | 7.5% |
| **Location** |  |  |
| Head of pancreas | 30148 | 54.5% |
| Body of pancreas | 7075 | 12.8% |
| Tail of pancreas | 6640 | 12.0% |
| Pancreatic duct | 331 | 0.6% |
| Other specified parts of pancreas | 823 | 1.5% |
| Overlapping lesion of pancreas | 4162 | 7.5% |
| Pancreas, NOS | 6139 | 11.1% |
| **Surgery** |  |  |
| No surgery | 43029 | 77.8% |
| Local or partial pancreatectomy | 1560 | 2.8% |
| Local or partial pancreatectomy and duodenectomy | 8417 | 15.2% |
| Total pancreatectomy with or without gastrectomy or duodenectomy | 2028 | 3.7% |
| Pancreatectomy NOS or surgery NOS | 284 | 0.5% |
| **Tumor size (cm)** |  |  |
| <=2 | 5027 | 9.1% |
| 2 to 4 | 23802 | 43.0% |
| 4 to 6 | 12721 | 23.0% |
| >6 | 3944 | 7.1% |
| Unknown | 9824 | 17.8% |
| **LNR** |  |  |
| 0 | 4679 | 8.5% |
| <=0.2 | 4109 | 7.4% |
| 0.2-0.4 | 2237 | 4.0% |
| 0.4-1 | 2171 | 3.9% |
| No nodes were examined | 42122 | 76.1% |
| **T stage** |  |  |
| T1-T2 | 12049 | 21.8% |
| T3-T4 | 34166 | 61.8% |
| Unknown | 9103 | 16.5% |
| **N stage** |  |  |
| NO | 27448 | 49.6% |
| N1 | 19909 | 36.0% |
| Unknown | 7961 | 14.4% |
| **M stage** |  |  |
| M0 | 27152 | 49.1% |
| M1 | 26464 | 47.8% |
| Unknown | 1702 | 3.1% |
| **Grade** |  |  |
| I-II | 12265 | 22.2% |
| III-IV | 9011 | 16.3% |
| Unknown | 34042 | 61.5% |
| **Chemotherapy** |  |  |
| No | 20898 | 37.8% |
| Yes | 34420 | 62.2% |
| **Radiotherapy** |  |  |
| No | 43304 | 78.3% |
| Yes | 12014 | 21.7% |
| **Marital status** |  |  |
| Married | 32781 | 59.3% |
| Single | 22537 | 40.7% |
