# Supplementary material for: Prognostic analysis of very early onset pancreatic cancer: a population-based analysis
Source: PeerJ. 2020 Feb 10;8:e8412. doi: 10.7717/peerj.8412 (PMC7017800; doi:10.7717/peerj.8412)
Supplement: Table S2 [file peerj-08-8412-s002.docx]

**Supplemental Table 2. The SH model based nomogram scores for each included variable in current study**

| Characteristics | SH model based Nomogram scores |
| --- | --- |
| **Sex** |  |
| Male | 54 |
| Female | 46 |
| **Race** |  |
| Caucasian | 54 |
| African American | 56 |
| American Indian/Alaska Native | 56 |
| Asian or Pacific Islander | 82 |
| **Location** |  |
| Head of pancreas | 54 |
| Body of pancreas | 47 |
| Tail of pancreas | 58 |
| Pancreatic duct | 100 |
| Other specified parts of pancreas | 27 |
| Overlapping lesion of pancreas | 48 |
| Pancreas, NOS | 71 |
| **Surgery** |  |
| No surgery | 54 |
| Local or partial pancreatectomy | 0 |
| Local or partial pancreatectomy and duodenectomy | 8 |
| Total pancreatectomy with or without gastrectomy or duodenectomy | 2 |
| Pancreatectomy NOS or surgery NOS | 36 |
| **Tumor size (cm)** |  |
| <=2 | 54 |
| 2 to 4 | 60 |
| 4 to 6 | 62 |
| >6 | 70 |
| Unknown | 55 |
| **LNR** |  |
| 0 | 54 |
| <=0.2 | 72 |
| 0.2-0.4 | 87 |
| 0.4-1 | 90 |
| No nodes were examined | 70 |
| **T stage** |  |
| T1-T2 | 54 |
| T3-T4 | 63 |
| Unknown | 50 |
| **N stage** |  |
| NO | 54 |
| N1 | 62 |
| Unknown | 74 |
| **M stage** |  |
| M0 | 54 |
| M1 | 78 |
| Unknown | 58 |
| **Grade** |  |
| I-II | 54 |
| III-IV | 70 |
| Unknown | 60 |
| **Chemotherapy** |  |
| No | 54 |
| Yes | 27 |
| **Radiotherapy** |  |
| No | 54 |
| Yes | 60 |
| **Marital status** |  |
| Married | 54 |
| Single | 63 |
