# Supplementary material for: Prognostic analysis of very early onset pancreatic cancer: a population-based analysis
Source: PeerJ. 2020 Feb 10;8:e8412. doi: 10.7717/peerj.8412 (PMC7017800; doi:10.7717/peerj.8412)
Supplement: Table S3 [file peerj-08-8412-s003.docx]

**Supplemental Table 3. The demographic and treatment data of patients with or without surgery**

| Characteristics |  | With surgery (N = 12289) | | | | |  | Without surgery (N = 43029) | | | | |
| --- | --- | --- | --- | --- | --- | --- | --- | --- | --- | --- | --- | --- |
|  |  | VEOPC (N = 346) | |  | Older PC (N = 11943) | |  | VEOPC (N = 1040) | |  | Older PC (N = 41989) | |
|  |  | No. of patients | % |  | No. of patients | % |  | No. of patients | % |  | No. of patients | % |
| T stage |  |  |  |  |  |  |  |  |  |  |  |  |
| T1-T2 |  | 67 | 19.4% |  | 2100 | 17.6% |  | 208 | 20.0% |  | 9674 | 23.0% |
| T3-T4 |  | 268 | 77.5% |  | 9675 | 81.0% |  | 597 | 57.4% |  | 23626 | 56.3% |
| Unknown |  | 11 | 3.2% |  | 168 | 1.4% |  | 235 | 22.6% |  | 8689 | 20.7% |
| N stage |  |  |  |  |  |  |  |  |  |  |  |  |
| NO |  | 112 | 32.4% |  | 4156 | 34.8% |  | 491 | 47.2% |  | 22689 | 54.0% |
| N1 |  | 230 | 66.5% |  | 7689 | 64.4% |  | 354 | 34.0% |  | 11636 | 27.7% |
| Unknown |  | 4 | 1.2% |  | 98 | 0.8% |  | 195 | 18.8% |  | 7664 | 18.3% |
| M stage |  |  |  |  |  |  |  |  |  |  |  |  |
| M0 |  | 312 | 90.2% |  | 11082 | 92.8% |  | 270 | 26.0% |  | 15488 | 36.9% |
| M1 |  | 32 | 9.3% |  | 755 | 6.3% |  | 754 | 72.5% |  | 24923 | 59.4% |
| Unknown |  | 2 | 0.6% |  | 106 | 0.9% |  | 16 | 1.5% |  | 1578 | 3.8% |
| Chemotherapy | |  |  |  |  |  |  |  |  |  |  |  |
| No |  | 73 | 21.1% |  | 3563 | 29.8% |  | 239 | 23.0% |  | 17023 | 40.5% |
| Yes |  | 273 | 78.9% |  | 8380 | 70.2% |  | 801 | 77.0% |  | 24966 | 59.5% |
| Radiotherapy | |  |  |  |  |  |  |  |  |  |  |  |
| No |  | 199 | 57.5% |  | 7392 | 61.9% |  | 822 | 79.0% |  | 34891 | 83.1% |
| Yes |  | 147 | 42.5% |  | 4551 | 38.1% |  | 218 | 21.0% |  | 7098 | 16.9% |
