# Supplementary figures and images for: Prognostic analysis of very early onset pancreatic cancer: a population-based analysis
Source: PeerJ. 2020 Feb 10;8:e8412. doi: 10.7717/peerj.8412 (PMC7017800; doi:10.7717/peerj.8412)

**Supplemental Figure 1.** The data selection process of current study


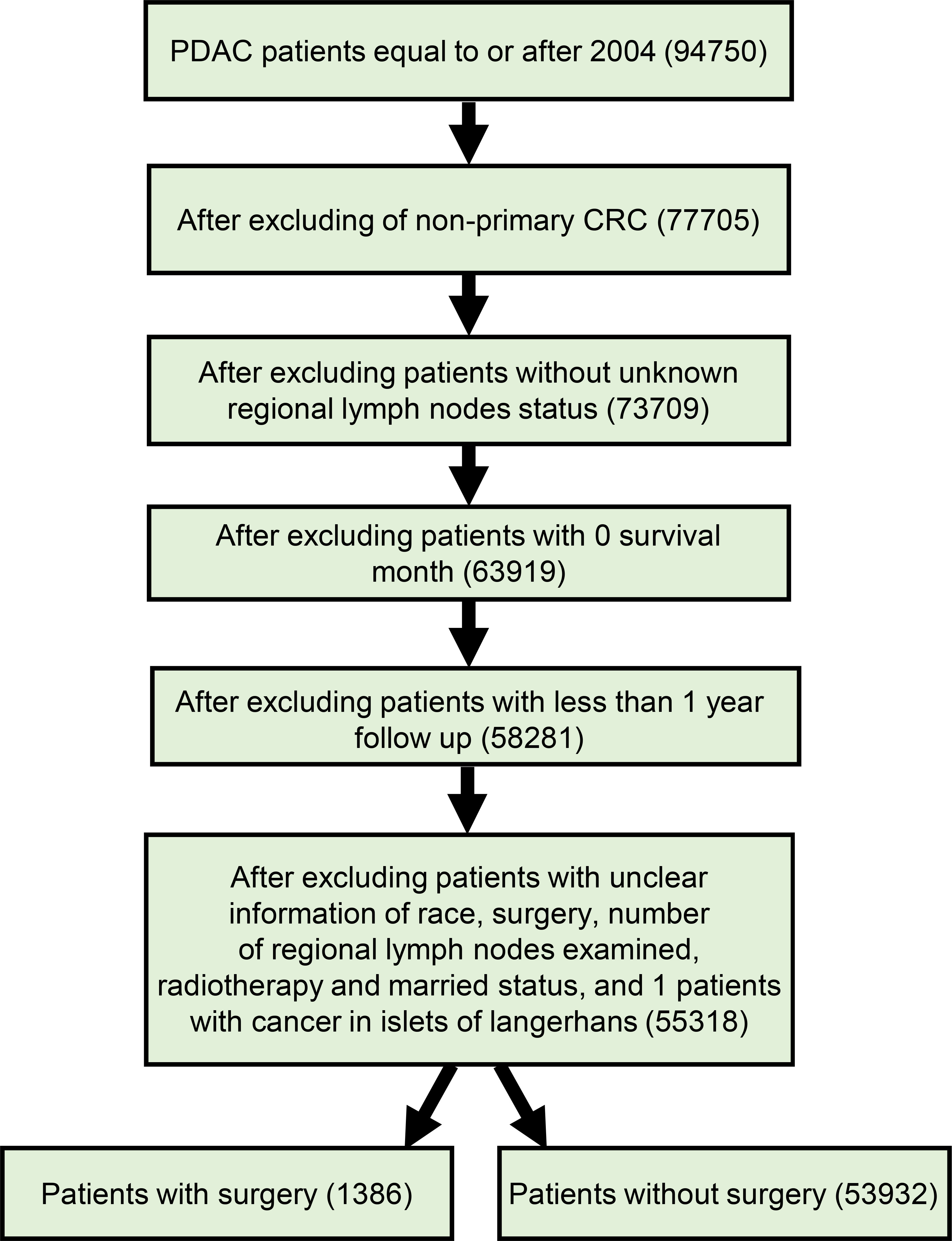

Supplement: Figure S1 [file peerj-08-8412-s004.docx]
